# Ferumoxytol Labeling of Human Neural Progenitor Cells for Diagnostic Cellular Tracking in the Porcine Spinal Cord with Magnetic Resonance Imaging

**DOI:** 10.5966/sctm.2015-0422

**Published:** 2016-08-29

**Authors:** Jason J. Lamanna, Juanmarco Gutierrez, Lindsey N. Urquia, C. Victor Hurtig, Elman Amador, Natalia Grin, Clive N. Svendsen, Thais Federici, John N. Oshinski, Nicholas M. Boulis

**Affiliations:** ^1^ Department of Neurosurgery, School of Medicine, Emory University, Atlanta, Georgia, USA; ^2^ Department of Biomedical Engineering, Georgia Institute of Technology and Emory University, Atlanta, Georgia, USA; ^3^ Board of Governors Regenerative Medicine Institute, Cedars‐Sinai Medical Center, Los Angeles, California, USA; ^4^ Department of Radiology and Imaging Sciences, School of Medicine, Emory University, Atlanta, Georgia, USA

**Keywords:** Cell transplantation, Clinical translation, In vivo tracking, Neural stem cell, Pig model, Stem cell transplantation, Magnetic resonance imaging, Spinal cord

## Abstract

We report on the diagnostic capability of magnetic resonance imaging (MRI)‐based tracking of ferumoxytol‐labeled human neural progenitor cells (hNPCs) transplanted into the porcine spinal cord. hNPCs prelabeled with two doses of ferumoxytol nanoparticles (hNPC‐F^Low^ and hNPC‐F^High^) were injected into the ventral horn of the spinal cord in healthy minipigs. Ferumoxytol‐labeled grafts were tracked in vivo up to 105 days after transplantation with MRI. Injection accuracy was assessed in vivo at day 14 and was predictive of “on” or “off” target cell graft location assessed by histology. No difference in long‐term cell survival, assessed by quantitative stereology, was observed among hNPC‐F^Low^, hNPC‐F^High^, or control grafts. Histological iron colocalized with MRI signal and engrafted human nuclei. Furthermore, the ferumoxytol‐labeled cells retained nanoparticles and function in vivo. This approach represents an important leap forward toward facilitating translation of cell‐tracking technologies to clinical trials by providing a method of assessing transplantation accuracy, delivered dose, and potentially cell survival. Stem Cells Translational Medicine *2017;6:139–150*

**Significance Statement**

This is the first report to document the diagnostic capabilities of iron oxide nanoparticle‐labeled cells in the central nervous system of a large animal model. The major findings of this study were (a) the diagnostic capability of in vivo magnetic resonance imaging for evaluating ferumoxytol‐labeled cell graft injection accuracy and initial volume; (b) the long‐term survival of ferumoxytol‐labeled cells in a large animal model; and (c) postmortem confirmation that ferumoxytol‐labeled cells retain nanoparticles and function in vivo. The use of a U.S. Food and Drug Administration (FDA)‐approved iron oxide nanoparticle, ferumoxytol, in combination with research‐grade human neural progenitor cells, a large animal transplantation model, and a clinical magnetic resonance scanner make this study immediately applicable to clinical investigation and informative to FDA Investigational New Drug enabling applications.

## Introduction

Stem cell transplantation into the spinal cord presents a promising therapeutic strategy to overcome the regenerative limitations of the central nervous system (CNS) in degenerative and traumatic pathologies. Cell transplantation has been investigated clinically for amyotrophic lateral sclerosis (ALS), multiple sclerosis, and traumatic spinal cord injury 1. Emerging evidence from these reports indicates that direct transplantation into the cord is safe, feasible, and well tolerated and may have therapeutic benefits 2, 3. However, assessing therapeutic efficacy has been complicated by the inability of clinical investigators to measure transplantation targeting accuracy, delivered dose, or cell survival because of ineffective postmortem histology and the lack of a diagnostic marker for identifying cell grafts.

Calculating targeting accuracy for individual patients is important because only cell grafts delivered “on” target will generate a therapeutic benefit (e.g., the ventral horn for ALS). Reliable targeting of the injection cannula to the ventral horn is based on preoperative imaging and visual observation of spinal cord anatomy but is complicated by spinal cord surface vasculature, its small size, and its relative depth in the cord. Knowledge of delivered cell dose and survival are also crucial. During transplantation, the only indication of successful graft injection is with observation in the operating room of movement of cell suspension in the cannula tubing, suggesting that the cell suspension is being injected into the spinal cord. The volume of cells actually delivered to the target site remains uncertain because delivery can be confounded by many factors, including cannula obstruction and reflux out of the spinal cord. The importance of confirming accurate therapeutic delivery was highlighted in early gene therapy studies in which improper dosing and unreliable delivery contributed to a lack of therapeutic efficacy in clinical trials 4, 5. These findings helped build the foundation for magnetic resonance (MR)‐guided delivery with molecular imaging markers to confirm accurate injection to deep brain structures 6, 7. Furthermore, it has been shown that survival of neural progenitor cells transplanted into the CNS is dependent on transplantation site, where cell grafts transplanted into white mater were rejected, whereas those in gray mater were accepted 8. Therefore, it is critical to develop and test diagnostic, noninvasive imaging technologies that allow for in vivo and postmortem cell graft assessment.

Previous groups have used a variety of molecular imaging strategies for monitoring cell therapies. Reporter gene systems have been designed for bioluminescence imaging 9, optical imaging 10, positron emission tomography (PET) 11, and MR imaging (MRI) 12. Exogenous contrast agents, including radionuclides for PET and superparamagnetic iron oxide nanoparticles (SPIONs) for MRI, have been used to physically label cells prior to transplantation and represent a less invasive alternative to reporter gene systems 13, 14. SPIONs are under investigation as a molecular imaging contrast agent for labeling and tracking transplanted cells with MRI 15, 16. SPIONs are nanometer‐sized particles with a biodegradable carbohydrate coat and an iron oxide core capable of generating robust contrast to distinguish transplanted cells from surrounding host tissue using in vivo MRI and postmortem histology. These approaches have been explored clinically in both the brain 17, 18 and spinal cord 19, 20. Recently, ferumoxytol, an ultrasmall SPION that has U.S. Food and Drug Administration (FDA) approval for the treatment of iron‐deficiency anemia in chronic kidney disease, has been explored as a new agent for cell tracking. Ferumoxytol alone or in combination with transfection agents protamine, heparin, or both has been shown to be an effective method for labeling cells and tracking them in small animal models 21, 22.

The purpose of this study was to transplant ferumoxytol‐labeled cells into the spinal cord of a large animal (minipigs) and use ferumoxytol as a noninvasive diagnostic marker. In this report, we demonstrate the ability to evaluate cell graft injection accuracy noninvasively, in vivo with MRI, calculate graft volume in vivo, and estimate graft survival with postmortem histology with a diagnostic marker that does not alter long‐term cell graft survival or function. The cell tracking was conducted with a clinical 3.0 Tesla MR scanner in a large animal to assess the ability to track stem cell grafts in vivo in a model that is directly translatable to clinical trials.

## Materials and Methods

### Cell Culture and Preparation

Frozen stocks of early passage 21 human fetal cortex‐derived neural progenitor cells (hNPCs) were kindly provided by the Clive Svendsen laboratory at Cedars‐Sinai Regenerative Medicine Institute 23, 24. The hNPCs were originally isolated from 8‐week‐old postmortem fetal cortex of an aborted fetus with institutional review board approval. Briefly, the intact cortical mantel was isolated and dissociated to a single cell suspension. The resulting cell line was expanded to free‐floating neurospheres of hNPCs, and at passage 21 were frozen and sent to Emory University for the following studies.

The hNPCs were thawed and maintained as free‐floating neurospheres in T75 tissue culture flasks maintained with Neural Stem Cell Medium (Stemline Neural Stem Cell Expansion Medium, S3194; Sigma‐Aldrich, St. Louis, MO, https://www.sigmaaldrich.com), supplemented with recombinant human leukemia inhibitory factor (10 ng/ml, LIF1010; MilliporeSigma, Darmstadt, Germany, http://www.emdmillipore.com), recombinant human epidermal growth factor (100 ng/ml, GF003‐AF; MilliporeSigma), and antimicrobial/bacterial reagent (15240062; Thermo Fisher Scientific, Waltham, MA, https://www.thermofisher.com) (maintenance medium). The cells were cultured in a standard cell culture incubator at 37°C and 5% CO_2_. When the diameter of >75% of the neurospheres exceeded 500 µm, the neurospheres were passaged by mechanical sectioning 25. Briefly, one flask of neurospheres were isolated from the media, placed in a plastic petri dish, orthogonally sectioned with an automatic tissue chopper (McIlwain Tissue Chopper; Lafayette Instrument Co., Lafayette, IN, http://www.lafayetteinstrument.com), and split into two flasks with 50% fresh and 50% used maintenance media. The resulting clumps of cells reform spheres during the course of several days. The spheres were passaged approximately every 8 days, and 50% of the maintenance media were replaced with fresh maintenance media every 4 days. To prepare for transplantation and cytotoxicity assays, we chemically dissociated the neurospheres with trypsin (TrypLE Express, 12604; Thermo Fisher) and DNAse (D4527; MilliporeSigma) and filtered with a 50‐μ separation filter (130‐041‐407; Miltenyi Biotech) to a single cell suspension in magnesium‐ and calcium‐free hibernation medium (proprietary, provided by Svendsen Laboratory). The cells were concentrated to 1 × 10^4^ cells/μl and stored on ice. Cells between passages 25 and 35 were used in this study.

### Cell Labeling

The hNPCs cultured as free‐floating neurospheres were mechanically passaged when the mean diameter exceeded ∼500 μm 25. The chopped neurospheres were incubated with ferumoxytol nanoparticles (Ferraheme; AMAG Pharmaceuticals, Waltham, MA, http://www.amagpharma.com) of multiple doses ([0], [100], hNPC‐F^Low^ low dose [200], high dose hNPC‐F^High^ [400], or [1,000] μg/ml ferumoxytol) in maintenance medium for 168 hours. Fresh media with ferumoxytol nanoparticles replaced 50% of the conditioned media after 84 hours. The reformed neurospheres were dissociated, filtered to a single cell suspension, and concentrated to 1 × 10^4^ cells/μl for assessment and transplantation.

### Cell Viability

Trypan blue exclusion assay, flow cytometry, and a 3‐(4,5‐dimethylthiazol‐2‐yl)‐2,5‐diphenyltetrazolium bromide (MTT) assay were used to assess potential cytotoxicity of ferumoxytol‐labeled cells immediately after labeling. Quantitative flow cytometry live/dead staining (L34957; Thermo Fisher) was performed on the LSRFortessa flow cytometer. Gating and quantification was performed using FlowJo software. A MTT colorimetric assay (MTT Cell Growth Assay Kit, CT02; Sigma‐Aldrich) measuring mitochondrial metabolism was performed on cells attached to coated coverslips 24 hours after labeling. Briefly, MTT solution was added to the wells containing coverslips and incubated for 4 hours at 37°C for cleavage of MTT. The coverslips were then incubated overnight at 37°C with solubilization solution (10% sodium dodecyl sulfate in 0.01 M HCl). Absorbance was measured for each well at 570 nm (test) and 630 nm (reference) using an enzyme‐linked immunosorbent assay plate reader. Wells containing only media were used as background control, and wells containing dead hNPCs were used as a negative control.

### Flow Cytometry

Flow cytometry quantification of antigenic surface markers were quantified and compared with control cells. The cells were processed with the following antibodies: anti‐human leukocyte antigen (HLA)‐DR allophycocyanin (340549; BD Biosciences, East Rutherford, NJ, https://www.bdbiosciences.com), anti‐β2 microglobulin FITC (551338; BD Biosciences), anti‐CD80 PE (560925; BD Biosciences), and anti‐CD86 PE (560957; BD Biosciences). The samples were run on the LSRFortessa flow cytometer. Gating and quantification was performed using FlowJo software.

### In Vitro Histology

After incubation with ferumoxytol, cells were plated on laminin‐poly‐L‐ornithine‐coated glass coverslips for 24 hours, fixed with 4% paraformaldehyde (PFA), and Prussian Blue (PB) staining was performed to detect iron‐positive hNPCs, as has been previously described 26. Labeling efficiency was calculated with light microscopy of cells that were PB positive for intracellular iron nanoparticles and expressed as a percentage of positive cells per five high‐power fields. Plated cells were cultured in differentiation medium (Stemline medium [S3194; Sigma Aldrich]), supplemented with 2% B‐27 (17504044; Thermo Fisher) and antimicrobial/bacterial reagent (15240062; Thermo Fisher) for 7 days, fixed; immunofluorescence for glial fibrillary acidic protein (GFAP) (ab7260; Abcam, Cambridge, MA, http://www.abcam.com; 1/1000) and β‐tubulin III (T8660; Sigma‐Aldrich; 1/1000) was performed to detect differentiation of hNPCs, as has been previously described 25. Slides were stained with fluorophore‐coupled secondary antibodies (GAM488 and GAR594; Thermo Fisher; 1/500) and counterstained with 4′,6‐diamidino‐2‐phenylindole (DAPI) (Vectashield H‐1200; Vector Laboratories, Burlingame, CA, https://vectorlabs.com). Differentiation capacity was performed with fluorescent microscopy on cells that were GFAP or β‐tubulin III positive, expressed as a percentage of cells per five high‐power fields.

### Intracellular Iron Content

To quantify the amount of intracellular ferumoxytol, we performed a Perl's colorimetric assay of iron concentration 27. Briefly, aliquots of cells were lysed and mineralized with a high concentration of hydrochloric acid. Perl's reagent was added to generate a blue solution color, dependent on iron concentration, and absorbance at 630 nm was measured with an automated plate reader. The absorption of light from the cell samples was compared with a standard concentration curve of ferumoxytol particles. The absorbance of the cell sample was normalized to unlabeled cells and plotted on the standard curve to reveal the amount of iron from ferumoxytol particles in individual cells (pico‐grams per cell).

### Transplantation

A three‐level T_14_ to L_2_ laminectomy was performed in the thoracolumbar spine of 13 healthy female Göttingen minipigs. An incision was made into the dura mater, and three spinal cord segments were exposed. A stereotactic injection platform was used to insert a 29‐gauge injection needle into the spinal cord, as has been previously described 28. Twelve 25‐μl injections (10,000 cells/μl for 2.5 × 10^5^ cells/injection) were bilaterally infused at 5 μl per minute: four injections of hNPC‐F^High^ cell grafts into the rostral spinal cord segment, four hNPC grafts into the middle segment, and four hNPC‐F^Low^ grafts into the caudal segment of 11 minipigs. Two additional ferumoxytol nanoparticle‐only control minipigs received eight 25‐µl injections: four high‐dose ferumoxytol nanoparticle‐only injections (F^High)^ into the rostral spinal cord segment and four low‐dose ferumoxytol nanoparticle‐only injections (F^Low^) into the caudal segment. The low and high doses contained 3.65 × 10^−7^ g and 7.30 × 10^−7^ g of ferumoxytol nanoparticles in transplantation medium, which were equivalent to the amount of ferumoxytol in hNPC‐F^Low^ and hNPC‐F^High^ cell grafts, respectively. Intergraft spacing was 4 mm. Minipigs were immunosuppressed with tacrolimus (Prograf, 0.025 mg/kg, b.i.d., i.v.) for 28 days and then maintained on cyclosporine (Sandimmune, 10 mg/kg, b.i.d., oral) until euthanasia.

### Magnetic Resonance Imaging

Cellular and minipig MRI were performed on a clinical Siemens Tim Trio 3.0 Tesla whole body MRI scanner with a table‐integrated spine matrix coil (Siemens Medical Solutions, Malvern, PA, https://usa.healthcare.siemens.com). Structural images were obtained using standard sagittal T2‐weighted and coronal T1‐weighted three‐dimensional (3D) turbo spin echo sequences. T2* is the decay in transverse magnetization due to spin‐spin relaxation (T2), but in the setting of magnetic field inhomogeneity (gradient echo [GRE]). For labeled cell detection, a GRE T2*‐weighted axial sequence (multiple echo time = 10/16 ms, pulse repetition time = 788 ms, flip angle = 30°, averages = 4, field of view = 160 × 160 mm, matrix = 512 × 512, and slice thickness = 1.5 mm) was acquired twice (half‐slice thickness shift in second acquisition). Scans were performed preoperatively and following transplantation on days 14, 28, 42, 63, 84, and 105. Coronal reconstructions were made with 3D Slicer software ( http://www.slicer.org) 29.

### Magnetic Resonance Imaging Analysis

The volume of the individual grafts was calculated using ImageJ, adapting a previously described method for quantifying SPION signal in the rodent brain 30. Briefly, regions of interest in GRE MR images were set over half the spinal cord of individual hNPC‐F^Low^ and hNPC‐F^High^ grafts, encompassing the entire graft. Regions of interest over the entire cord were set in areas containing control hNPCs. For these control regions, a value for the average voxel intensity minus 2 SD was calculated. This value was used as a threshold for the regions containing labeled cells. The number of voxels below the threshold was calculated for each graft and was recorded in volume (microliters).

Anatomical position of each graft was determined by observing the distance anterior/posterior and left/right from the center of the spinal cord. Anatomical landmarks such as the gray/white junction and location of cerebrospinal fluid were also used. Three blinded expert observers viewed 20 grafts and scored them as “on” or “off” target. “On” target was defined as greater than 50% of the graft contacting the ventral horn. These data were used to determine on/off target transplantation using a chi‐square table to generate sensitivity and specificity for both hNPC‐F^Low^ and hNPC‐F^High^ grafts.

### Histopathology

Pigs were euthanized, perfused with heparinized 0.9% saline solution, fixed with 4% PFA, and the spinal cords excised. Pigs were euthanized on postoperative days 28 (*n* = 5), 42 (*n* = 3), and 105 (*n* = 5). The cords were placed in 4% PFA for an additional 24 hours and sucrose for 7 days, flash frozen, transaxially sectioned at 50 μm, and stored. Immunohistochemical staining for detection of grafted human cells using a primary mouse monoclonal anti‐human nucleus (HuNu) (MAB1281; Sigma‐Aldrich; 1/250) antibody was performed on every sixth section with cresyl violet background stain. Cell grafts were individually identified, and the number of grafted immunoreactive cells was estimated by using a stereological unbiased approach. Cell distribution was calculated with linear measurements in the principle axes of the transplanted cell grafts. For detection of histological iron, PB staining with Eosin background was performed on every 12th section in the region of grafted cells. Volume of histological iron was calculated graft‐wise using an ImageJ color threshold. To assess particle location and percentage of labeled cells, we performed PB‐HuNu costaining at the graft centers. Immunofluorescence staining with a primary mouse monoclonal anti‐human GFAP antibody (STEM123; Takara‐Bio, Saint‐Germain‐en‐Laye, France, http://www.takara-bio.com; 1/1000) and a rabbit polyclonal anti‐human nestin antibody (ABD69; Sigma‐Aldrich; 1/5000) was performed to assess graft differentiation and expressed as a relative percentage of cells per five high‐power fields. Proliferation was assessed with HuNu/Ki67 (ab15580; Abcam; 1/500) costain and apoptosis with HuNu/Cleaved Caspase 3 (9661; Cell Signaling Technology, Danvers, MA, https://www.cellsignal.com; 1/125) costain. Sections were stained with fluorophore‐coupled secondary antibodies (GAM488 and GAR594; 1/500) and counterstained with DAPI. Relative percentage of cells was calculated in 40× high‐powered fields with an ImageJ cell counter. Microglial activation was assessed with a rabbit polyclonal anti‐Iba1 antibody (019‐19741; Wako, Tokyo, Japan, http://www.wako-chem.co.jp/english; 1/250) with cresyl violet background stain. Images were captured with a digital DS‐Qi1 high‐sensitivity cooled CCD camera using a Nikon E400 microscope supplied with NIS‐Elements imaging software (Nikon Instruments, Inc., Tokyo, Japan, http://www.nikon.com). Stereology was performed with a microscope (DM2500; Leica, Wetzlar, Germany, https://us.leica-camera.com) with a motorized x–y stage, an electronic microcator (Applied Scientific Instrumentation, Eugene, OR, http://www.asiimaging.com), which was used for measuring movements in the *z* direction, and the PC software Stereologer for cell counting.

### Transmission Electron Microscopy

Frozen sections of 50 μm were thawed and washed thoroughly with 0.1‐M phosphate buffer to rinse cryoprotectant. The sections were incubated in blocking solution of phosphate‐buffered saline (PBS) containing 5% normal goat serum, 5% bovine serum albumin (BSA), and 0.1% cold‐water fish gelatin for 30 minutes at 4**°**C. Sections were then incubated in HuNu diluted with PBS containing 0.1% acetylated BSA (BSA‐c) to 5 µg/ml overnight at 4**°**C with gentle agitation. After six washes (5 minutes) with PBS/BSA‐c, sections were incubated overnight at 4**°**C in biotinylated secondary antibody (Vector; 1/200). After three washes with PBS/BSA‐c and three washes with PBS, sections were incubated in avidin‐biotin complex from ABC kit (Vector) for 3 hours and washed six times with PBS. Sections were placed in 0.05‐M Tris buffer (pH 7.3) containing 0.05% diaminobenzidine and 0.003% hydrogen peroxide for 5–10 minutes at room temperature. Sections were then washed, fixed with 2.5% glutaraldehyde in 0.1‐M PB, and embedded in Eponate 12 resin. Ultrathin sections were cut at 70 nm thick using a Leica UltraCut S ultramicrotome, counterstained with 5% uranyl acetate and 2% lead citrate, and examined on an JEOL JEM‐1400 transmission electron microscope (JEOL, Tokyo, Japan, http://www.jeol.co.jp/en) equipped with a Gatan 2k × 2k US1000 CCD camera (Gatan, Pleasanton, CA, http://www.gatan.com/).

### Statistical Analysis

Statistical analyses were performed using a standard one‐way analysis of variance (ANOVA) with Tukey's post hoc multiple comparisons. Linear regression and correlation analyses were done with a one‐sided *p* value (*p* < .05; *p* < .005; *p* < .0005). GraphPad prism software was used to determine significance and generate graphs. Each experiment was done in triplicate, at a minimum. Data are shown as mean ± SD.

## Results

### Cell Labeling and Iron Content

Human cortical neurospheres were incubated with increasing concentrations (0, 100, 200, 400, and 1,000 μg/ml) of ferumoxytol nanoparticles for 168 hours, following mechanical passage ( supplemental online Fig. 1A). The neurospheres were dissociated to a single cell suspension of hNPCs, and no significant difference in cell viability was observed ( supplemental online Fig. 1B). T2*‐weighted MRI of 2.5 × 10^5^ cell pellets showed a significant increase in negative T2* contrast with increased ferumoxytol dose ( supplemental online Fig. 1C, 1D). Incubation concentrations of low dose (200 μg/ml) and high dose (400 μg/ml) ferumoxytol were chosen for further evaluation (hNPC‐F^Low^ and hNPC‐F^High^).

**Figure 1:**
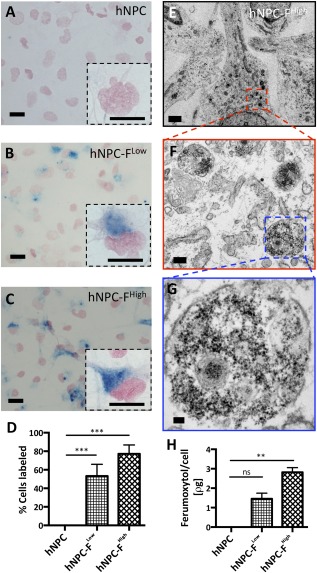
Cellular internalization of ferumoxytol nanoparticles. Shown are representative light microscopy images of cytochemical staining for cellular iron with Prussian Blue (PB), with Nuclear Fast Red counterstain, for unlabeled human neural progenitor cells (hNPCs) **(A)**, hNPC‐ ferumoxytol (F)^Low^‐labeled cells **(B)**, and hNPC‐F^High^‐labeled cells **(C)**, with high‐magnification images (insets). Characteristic blue precipitates of iron oxide nanoparticles were observed in the cytoplasm, adjacent to the nuclei of ferumoxytol‐labeled cells, and the percentage of labeled cells was quantified with ImageJ. A significant difference was observed between all groups with 53.3% and 77.2% of hNPC‐F^Low^ and hNPC‐F^High^ cells, respectively, labeled with ferumoxytol **(D)**. Transmission electron microscopy of hNPC‐F^Low^ and hNPC‐F^High^ cells **(E)** revealed numerous iron‐laden, electron‐dense endosomes **(F)** containing nanoparticles **(G)**. The cellular concentration of iron from ferumoxytol nanoparticles was calculated with a PB colorimetric digestion assay. A significant difference was observed with 1.46 and 2.82 ρg ferumoxytol iron/cell for hNPC‐F^Low^ and hNPC‐F^High^, respectively **(H)**. Scale bars = 10 μm, ×40 **(A–C)**; 10 μm, ×100 (insets); 3 μm **(E)**; 0.3 μm **(F)**; and 50 nm (**G**). Graphs are displayed as mean ± SD. ∗∗, significant at *p* < .005; ∗∗∗, significant at *p* < .0005. Abbreviations: F, ferumoxytol; hNPC, human neural progenitor cell; ns, not significant.

Of hNPC‐F^Low^ and hNPC‐F^High^ cells, 53.3% and 77.2% were Prussian Blue (PB) positive on histology (Fig. 1A–1D). Ferumoxytol nanoparticle internalization was confirmed with transmission electron microscopy (TEM), with electron‐dense nanoparticles of ∼10 nm in diameter observed in endosomes (Fig. 1E–1G). Particles were not observed on the cell membrane or in control cells. The average iron content per cell from ferumoxytol was 1.46 ± 0.29 pg/cell and 2.82 ± 0.24 pg/cell for hNPC‐F^Low^ and hNPC‐F^High^, respectively (Fig. 1H). To estimate the longevity of intracellular iron, hNPC‐F^Low^ and hNPC‐F^High^ were cultured for an additional 7 days, and intracellular electron‐dense nanoparticles were observed with TEM ( supplemental online Fig. 2).

### Ferumoxytol‐Labeled Cells: Viability and Functionality

**Figure 2:**
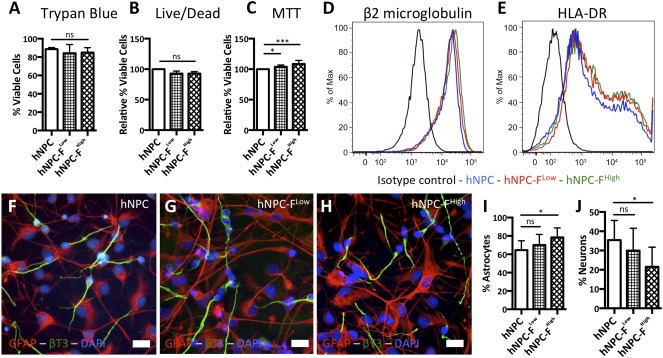
Cell viability and functionality in vitro following ferumoxytol labeling. Trypan Blue exclusion assay **(A)** and flow cytometry live/dead stain with AquaBlue **(B)** showed no change in cell viability among groups. MTT cellular metabolism assay showed a significant increase in cellular metabolism with ferumoxytol labeling **(C)**. Flow cytometry histogram plots of cellular antigens β2 microglobulin **(D)** and human leukocyte antigen‐DR **(E)** show no change in expression with ferumoxytol labeling. Representative images of immunocytochemical staining of unlabeled human neural progenitor cells (hNPCs) **(F)**, hNPC‐ferumoxytol (F)^Low^‐labeled cells **(G)**, and hNPC‐F^High^‐labeled cells **(H)** expressing the astrocytic marker GFAP (red) and the neuronal marker βT3 (green). An increase in astrocytic differentiation with a concurrent decrease in neuronal differentiation was observed between hNPC and hNPC‐F^High^ conditions, but not between unlabeled hNPC and hNPC‐F^Low^ **(I, J)**. Scale bars = ×40, 10 μm **(F–H)**. Graphs are displayed as mean ± SD. ∗, significant at *p* < .05; ∗∗∗, significant at *p* < .0005. Abbreviations: βT3, beta III tubulin; DAPI, 49,6‐diamidino‐2‐phenylindole; F, ferumoxytol; GFAP, glial fibrillary acidic protein; HLA, human leukocyte antigen; HLA‐DR, human leukocyte antigen D related; hNPC, human neural progenitor cell; Max, maximum; MTT, 3‐(4,5‐dimethylthiazol‐2‐yl)‐2,5‐diphenyltetrazolium bromide; ns, not significant.

There were no substantial changes in cell viability measured by Trypan Blue or flow cytometry live/dead stain (Fig. 2A, 2B). Passaged neurospheres labeled with ferumoxytol reformed healthy neurospheres, and no changes in viability were observed in these cells 1 week after passage ( supplemental online Fig. 3). An increase (ANOVA; *p* < .0005) in mitochondrial metabolism (MTT) was observed with increased ferumoxytol dose in comparison with control cells (Fig. 2C). Phenotypic analysis of surface antigenicity markers with flow cytometry was positive for β2 microglobulin (MHC I) and HLA‐DR (MHC II), and no changes were observed with ferumoxytol labeling (Fig. 2D, 2E). The differentiation potential of hNPCs toward astrocytic and neuronal lineages showed a shift toward the astrocytic lineage in high‐dose hNPC‐F^High^ cells (Fig. 2F–2J). We observed >99% differentiation of attached cells in all groups.

### Transplantation and in Vivo MRI

**Figure 3:**
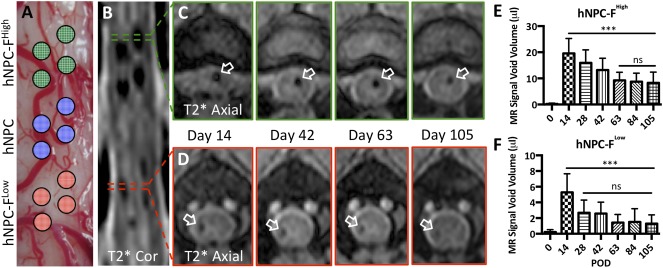
In vivo tracking and quantification of ferumoxytol‐labeled grafts in the porcine spinal cord at 3T. Individual grafts of unlabeled human neural progenitor cell (hNPC) and ferumoxytol‐labeled hNPC‐F^Low^ and hNPC‐F^High^ cells were bilaterally transplanted into the porcine spinal cord with a stabilized stereotactic injection system. hNPC‐F^High^ cell grafts were transplanted into the rostral spinal cord segment, unlabeled hNPC grafts into the center segment, and hNPC‐F^Low^ grafts were transplanted into the caudal segment for 12 total injections of 2.5 × 10^5^ cells each **(A)**. Hypointense foci, representative of hNPC‐F^Low^‐ and hNPC‐F^High^‐labeled cell grafts, were observed on postoperative day 14 in a representative coronal T2*‐weighted reconstruction **(B)**. Unlabeled cell grafts were not visualized. Representative axial T2*‐weighted images of hNPC‐F^High^ **(C)** and hNPC‐F^Low^ **(D)** grafts (white arrows) were tracked with serial magnetic resonance imaging for up to 105 days. The graft volumes were calculated for all hNPC‐F^High^ **(E)** and hNPC‐F^Low^ **(F)** grafts at all time points. Summary data represented as the mean signal intensity at each time point are shown. A significant decline in signal void volume was observed between day 14 and other time points, but no change was observed after day 63 for hNPC‐F^High^ grafts **(E)**. No significant change was observed after day 28 for hNPC‐F^Low^ grafts **(F)**. Graphs are displayed as mean ± SD. ∗∗∗, significant at *p* < .0005. Abbreviations: Cor, coronal; F, ferumoxytol; hNPC, human neural progenitor cell; MR, magnetic resonance; ns, not significant; POD, postoperative day.

Direct intraspinal transplantation of four hNPC‐F^High^ cell grafts into the rostral spinal cord segment, four hNPC grafts into the middle segment, and four hNPC‐F^Low^ grafts into the caudal segment (12 total injections of 2.5 × 10^5^ cells each 31) was performed in the thoracolumbar enlargement of 11 pigs (Fig. 3A). No permanent postoperative sensory or motor deficits were observed in experimental animals, and they returned to baseline at postoperative day 7 ( supplemental online Fig. 4). However, a postoperative deficit immediately following transplantation resulting in hind‐limb weakness was observed in one of the ferumoxytol nanoparticle‐only control animals, leading to early euthanasia 14 days after transplantation. Significant spinal canal stenosis with obliteration of anterior and posterior cerebrospinal fluid space was observed on T2‐weighted MRI 14 days after transplantation.

All hNPC‐F^Low^ and hNPC‐F^High^ transplanted cell grafts were identified with T2*‐weighted MRI as hypointense voxels at the injection sites, in comparison with surrounding parenchyma or unlabeled, control hNPC grafts 14 days after transplantation (Fig. 3B). The grafts were tracked with serial MRI for up to 105 days after transplantation (Fig. 3C, 3D). The average volume of hypointense voxels for hNPC‐F^Low^ and hNPC‐F^High^ cell grafts 14 days after transplantation was 5.3 ± 2.4 µl and 19.6 ± 5.7 µl, respectively (*p* < .0005). A significant decrease in hypointense voxel volume was observed over time for hNPC‐F^High^ (Fig. 3E) and hNPC‐F^Low^ grafts (Fig. 3F). The average volume at day 105 was 1.2 ± 1.1 µl, with 65% of hNPC‐F^Low^ cell grafts identified (Fig. 3F), and 8.3 ± 4.1 µl, with 100% of hNPC‐F^High^ grafts identified (Fig. 3E). A mean hypointense voxel volume decline of 58% and 77% was observed for hNPC‐F^Low^ and hNPC‐F^High^ cell grafts, respectively. The average volume of hypointense voxels for control injections F^Low^ and F^High^ 14 days after transplantation was 1.5 ± 1.5 µl and 12.5 ± 3.3 µl, respectively (*p* < .0005) ( supplemental online Fig. 5). The volume remained stable for F^Low^ at 28 days (1.7 ± 1.0 µl), but a decline was observed in F^High^ injections (3.7 ± 1.6 µl; *p* < .005).

**Figure 4:**
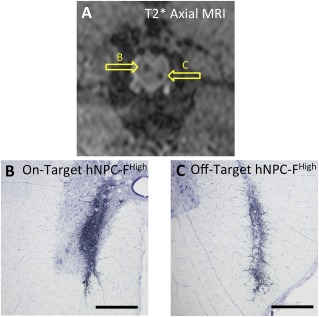
Diagnostic magnetic resonance image (MRI) of ferumoxytol‐labeled cell grafts is predictive of On/Off target transplantation site. A T2*‐weighted axial MR image **(A)** showed both an on‐target **(B)** and an off‐target **(C)** ferumoxytol‐labeled human neural progenitor cell (hNPC‐F)^High^ graft in the spinal cord (arrows, labeled to correspond to panels B and C). A representative “on target” **(B)** and “off target” **(C)** micrograph of hNPC‐F^High^ cell grafts stained for the human nuclear antigen (black nuclei) are shown. “On target” was defined as greater than 50% of the cell graft contacting the motor neuron‐containing ventral horn. Three blinded expert observers reviewed T2*‐weighted MR images of both hNPC‐F^Low^ and hNPC‐F^High^ cell grafts. Diagnostic review of T2*‐weighted MRI of hNPC‐F^Low^ grafts had an 86.7% sensitivity and a 93.3% specificity in predicting “on” or “off” target grafts. MRI of hNPC‐F^High^ cell grafts had an 86.7% sensitivity and an 80.0% specificity in predicting targeting. Scale bars = ×4, 1 mm. Abbreviations: F, ferumoxytol; hNPC, human neural progenitor cell; MRI, magnetic resonance imaging.

Blinded expert observers reviewed T2*‐weighted images of intraspinal hNPC‐F^Low^ and hNPC‐F^High^ cell grafts to determine whether each injection was “on” or “off” target (Fig. 4A). Histological confirmation of graft location was used as the gold standard (Fig. 4B, 4C). “On” target was defined as >50% of the transplanted cell graft contacting the ventral horn. Of all cell grafts, 54.8% ± 20.2% were “on” target with an interanimal range of 33.3% to 91.7%. Diagnostic localization of hNPC‐F^Low^ cell grafts had a sensitivity of 86.7% and specificity of 93.3%, and hNPC‐F^High^ grafts had an 86.7% sensitivity and 80.0% specificity in predicting “on” target transplantation location.

### Histological Identification

**Figure 5:**
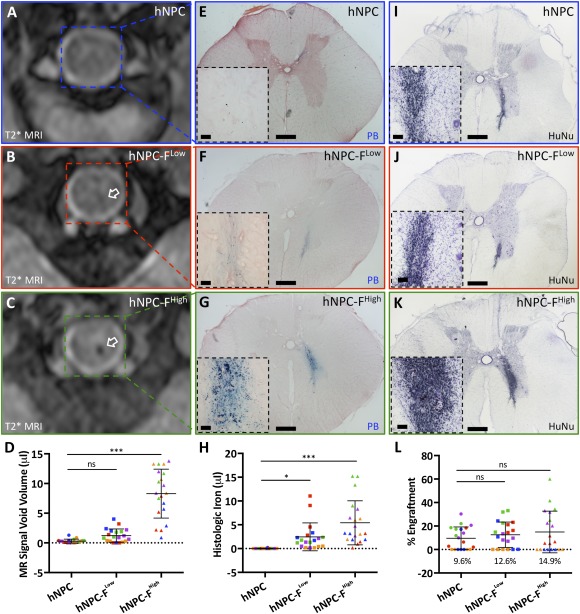
Ferumoxytol‐labeled cell grafts identified postmortem in the porcine spinal cord. A representative T2*‐weighted magnetic resonance (MR) image of the region containing a human neural progenitor cell (hNPC) unlabeled graft 105 days after transplantation showed no hypointense focus **(A)**. Representative MR images from representative ferumoxytol‐labeled hNPC (hNPC‐F)^Low^ **(B)** and hNPC‐F^High^ **(C)** cell grafts showed hypointense foci (white arrows), representative of the negative contrast produced by ferumoxytol. The MR signal void volume for all grafts was calculated for the terminal time point, 105 days **(D)**. Representative micrographs from Prussian Blue iron staining are shown for hNPC **(E)**, hNPC‐F^Low^ **(F)**, and hNPC‐F^High^ **(G)** cell grafts. Characteristic blue precipitates were observed in hNPC‐F^Low^ and hNPC‐F^High^ cell grafts. Graft‐specific histological iron was quantified **(H)**. Representative micrographs of human nuclear antigen staining for hNPC **(I),** hNPC‐F^Low^ **(J)**, and hNPC‐F^High^ **(K)** cell grafts are shown. Engraftment was quantified with stereology **(L)**. Scale bars = ×2, 1 mm; ×20, 50 μm (inset). Graphs displayed as mean ± SD. Individual data points on graphs D, H, and L are from individual grafts at postoperative day 105 and color coded by animal. ∗, significant at *p* < .05; ∗∗∗, significant at *p* < .0005. Abbreviations: F, ferumoxytol; hNPC, human neural progenitor cell; HuNu, human nucleus; MRI, magnetic resonance imaging; ns, not significant; PB, Prussian Blue.

T2*‐weighted MR images acquired immediately prior to euthanasia at postoperative day (POD) 105 (Fig. 5A–5D) colocalize well with corresponding Prussian Blue photomicrographs of hNPC‐F^Low^ and hNPC‐F^High^ cell grafts (Fig. 5E–5G). Significant histological iron deposits were observed and quantified for each cell graft (Fig. 5H). Histological iron deposits were not observed in unlabeled grafts. Corresponding human nucleus (HuNu)‐positive photomicrographs show colocalization of immunoreactive hNPC‐F^Low^ and hNPC‐F^High^ cell grafts with histological iron deposits (Fig. 5I–5K).

Stereological quantification of HuNu immunoreactive cells in hNPC (9.6% engraftment), hNPC‐F^Low^ (12.6%), and hNPC‐F^High^ (14.9%) grafts showed no difference in cell survival between groups 105 days after transplantation (Fig. 5L). Cell engraftment and colocalization with histological iron deposits remained stable during different time points POD28 and POD42 ( supplemental online Fig. 6). A linear correlation between cell engraftment and histologic iron deposits was observed with the hNPC‐F^High^ cell grafts (*r* = .63, *p* < .0005). However, it is important to note that histological iron was observed in all rejected hNPC‐F^Low^ and hNPC‐F^High^ cell grafts.

### Graft Characterization

**Figure 6:**
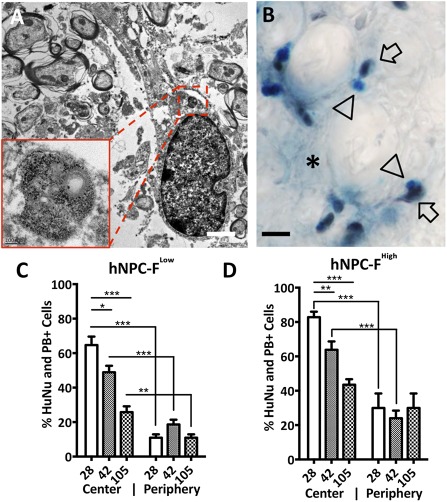
Ferumoxytol remains internalized by transplanted human neural progenitor cells. A representative image of transmission electron microscopy of a 3,3′‐diaminbenzidine (DAB)‐human nucleus positive, transplanted ferumoxytol‐labeled human neural progenitor cell (hNPC‐F^High^) **(A)** reveals numerous iron‐laden, electron dense endosomes (inset) at postoperative day 42. DAB‐human nucleus with Prussian Blue co‐staining reveals colocalization of iron precipitates (arrowheads) and human nuclei (arrows) **(B)** at postoperative day 105. Iron precipitates were also observed outside of human cells (asterisk), but with a lower density. Quantification of the percentage of HuNu+ cells colocalizing with PB+ precipitates was used to calculate the percentage of labeled hNPC‐F^Low^ **(C)** and hNPC‐F^High^ **(D)** cell grafts from postoperative days 28, 42, and 105. The “center” refers to the core of the cell graft and the “periphery” refers to cells migrating 200 μm or more from the graft core. Scale bars = 2 μm **(A)**; 100 nm (inset); and ×40, 25 μm **(B)**. ∗, significant at *p* < .05; ∗∗, significant at *p* < .005; ∗∗∗, significant at *p* < .0005. Abbreviations: F, ferumoxytol; hNPC, human neural progenitor cell; HuNu, human nuclei; PB, Prussian Blue.

**Figure 7:**
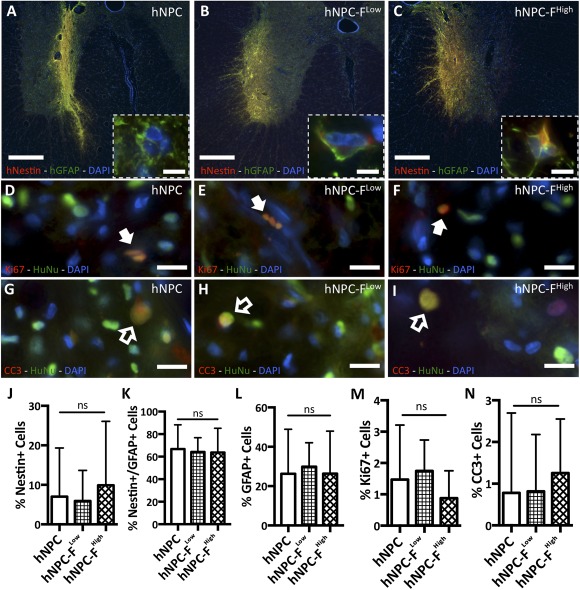
Transplanted ferumoxytol‐labeled human neural progenitor cells differentiate in the porcine spinal cord. Representative fluorescent micrographs of immunohistochemical staining with a mouse monoclonal anti‐human glial fibrillary acidic protein (GFAP) antibody (green) and a rabbit polyclonal anti‐human nestin antibody (red) in unlabeled human neural progenitor cells (hNPC) **(A)**, ferumoxytol‐labeled hNPC‐(F)^Low^ **(B)**, and hNPC‐F^High^ **(C)** grafts from postoperative day 105 are shown with high‐magnification insets. All nuclei are observed with 49,6‐diamidino‐2‐phenylindole staining (blue). Five grafts from each condition with >10% engraftment were chosen for analysis. GFAP+ or nestin+ signal was not observed on the contralateral side or in rejected grafts. High‐magnification images of unlabeled hNPC (**D**), hNPC‐F^Low^ (**E**), and hNPC‐F^High^ (**F**) cell grafts expressing human nucleus (HuNu+) (green) and Ki67+ (red) show few proliferating transplanted cells (solid arrows). High‐magnification images of unlabeled hNPC **(G)**, hNPC‐F^Low^ **(H)**, and hNPC‐F^High^ **(I)** cell grafts expressing HuNu+ (green) and cleaved caspase‐3 (CC3) (red) show few apoptotic transplanted cells (open arrows). The relative expression of GFAP+ and nestin+ was calculated for all groups **(J‐L)**. The relative expression of HuNu+ and Ki67+ was calculated **(M)**, as was that between HuNu+ and CC3 **(N)**. Scale bars = ×4, 500 μm **(A‐C)**; ×100, 10 μm **(A‐C)** (insets); ×40, 25 μm **(D‐I)**. Graphs are displayed as mean ± SD. Abbreviations: CC3, cleaved caspase 3; DAPI, 4′,6‐diamidino‐2‐phenylindole; F, ferumoxytol; GFAP, glial fibrillary acidic protein; hNPC, human neural progenitor cell; HuNu, human nucleus; ns, not significant.

Electron‐dense nanoparticles were observed in the endosomes of HuNu immunoreactive cells in hNPC‐F^Low^ and hNPC‐F^High^ grafts in vivo with TEM at POD42 (Fig. 6A). PB‐HuNu costaining revealed immunoreactive human nuclei colocalizing with iron precipitates at all time points (Fig. 6B). Quantification of HuNu+ and PB+ cells revealed a significant decline in the percentage of ferumoxytol‐labeled cells over time for both hNPC‐F^Low^ and hNPC‐F^High^ grafts (Fig. 6C, 6D). Furthermore, among cells migrating 200 µm or more from the core of the graft to the periphery, significantly fewer were both HuNu+ and PB+ (64.6% vs. 10.9% in hNPC‐F^Low^ and 82.2% vs. 30.0% in hNPC‐F^High^ grafts at POD28; *p* < .0005). Minimal changes in cell distribution were observed among hNPC, hNPC‐F^Low^, and hNPC‐F^High^ grafts ( supplemental online Fig. 7). At 105 days after transplantation, the cell grafts expressed a combination of nestin and GFAP. No difference was observed in the relative expression of GFAP and nestin in hNPC (7.0% ± 12.3% nestin+, 66.7% ± 21.5% nestin/GFAP+, 26.3% ± 22.7% GFAP+), hNPC‐F^Low^ (5.9% ± 7.7% nestin+, 64.2% ± 12.7% nestin/GFAP+, 29.9% ± 12.2% GFAP+), or hNPC‐F^High^ (9.9% ± 16.2% nestin+, 63.7% ± 21.4% nestin/GFAP+, 26.4% ± 21.6% GFAP+) cell grafts (Fig. 7A–7C, 7J–7L). Fewer than 2% of cells were Ki67+ (Fig. 7D–7F, 7M) or Cleaved Caspase 3+ (Fig. 7G–7I, 7N) for all groups. Furthermore, limited microglial activation was observed in accepted hNPC, hNPC‐F^Low^, and hNPC‐F^High^ grafts at POD105 ( supplemental online Fig. 8).

## Discussion

The major findings of this study were as follows: (a) the diagnostic capability of in vivo MRI for evaluating ferumoxytol‐labeled cell graft injection accuracy and initial volume; (b) the long‐term survival of ferumoxytol‐labeled cells in a large animal model; and (c) postmortem confirmation that ferumoxytol‐labeled cells retain nanoparticles and function in vivo. The use of an FDA‐approved iron oxide nanoparticle, ferumoxytol, in combination with research‐grade human neural progenitor cells, a large animal transplantation model, and a clinical MR scanner make this study immediately applicable to clinical investigation and informative to FDA Investigational New Drug enabling applications. Furthermore, the techniques learned in this study can be applied to multiple cell lines for different indications in the spinal cord.

Noninvasive imaging for diagnostic therapeutic monitoring will play a critical role in the widespread translation of cellular therapeutics to the CNS 32. The current state of monitoring relies on postmortem histological analysis 33 and intraoperative observation 34. Previous work has shown effective cellular labeling with different combinations of ferumoxytol 22 and visualized labeled cells in central nervous systems of small animals 21. Furthermore, SPION‐labeled cells have been safely used in international clinical studies in the brain 17, 18 and spinal cord 19, 20. However, their clinical use has been limited to simply acknowledging the appearance of transplanted labeled cells. Quantitative studies assessing graft size or injection accuracy have not been performed, in part because of limited preclinical work in large animal models.

This is the first report to document the diagnostic capabilities of SPION‐labeled cells in the CNS of a large animal model. Although SPION‐labeled cells have been tracked in the spinal cord of small animal models, these studies were of relatively short duration 35. In contrast, SPION‐labeled cells have been tracked for longer than 1 year in the brain of small animal models 36, 37. To the best of our knowledge, this is the longest report published to date regarding the spinal cord. Transplanted hNPC‐F^Low^ and hNPC‐F^High^ grafts were clearly visualized in the spinal cord of the pig with 3T MRI 2 weeks after transplantation and observed up to 105 days after transplantation. The graft volume quantified with MRI corresponded with labeling condition and diminished over time. Control, ferumoxytol‐only injections had a smaller initial volume with more rapid dissemination and clearance from the spinal cord in comparison with ferumoxytol‐labeled hNPCs. This is likely due to rapid clearance by a combination of phagocytic cells and perivascular flow 38.

Analysis of initial posttransplantation MRI to determine location of hNPC‐F^Low^ and hNPC‐F^High^ grafts by expert observers was predictive of histological graft location of “on” or “off” target to the ventral horn. Knowledge of graft location could allow clinicians to gauge delivered dose to clinical trial patients on the basis of the percentage of cell grafts delivered “on” target. This is especially important considering that a range of 33.3% to 91.7% of cell grafts were delivered “on” target in this study, and there is a theoretical correlation between “on” target grafts and potential therapeutic benefit. It is important to note that the “on” target percentage is likely higher in clinical studies because of the use of more advanced surgical technologies.

The primary objective of this study was to quantitatively assess the utility of ferumoxytol labeling as a diagnostic cellular marker in the large animal spinal cord. No difference in long‐term, in vivo cell survival was observed between either ferumoxytol‐labeled hNPC‐F^Low^ or hNPC‐F^High^ grafts and unlabeled control hNPC grafts. Cell survival was in line with previous reports 31. Furthermore, no change in graft distribution, differentiation to terminal cell types, apoptosis, or proliferation was observed at 105 days’ posttransplantation for all conditions, suggesting that the labeled cell grafts retained a level of function. This evidence suggests that there was no long‐term in vivo cytotoxicity from potential cell‐nanoparticle interactions. Increased astrocytic differentiation was observed in vitro with hNPC‐F^High^ cells. The mechanism underlying this shift in differentiation fate is unknown, but changes in differentiation for other cell lines have been previously observed with SPION labeling 39, 40. However, it is important to note that this cell line has an inherited potential to differentiate into astrocytes 24. The current study was not designed to investigate the therapeutic efficacy of the transplanted cell graft, which has been established in previous studies 23, 24, 41.

The cell‐tracking method presented here provides insight into the initial volume and location of transplanted grafts, but it is difficult to draw conclusions about long‐term graft survival using this approach. The decline in MR signal over time is most likely due to a combination of transplanted cell death with clearance of SPION by phagocytic cells and exocytosis of SPION by surviving transplanted cells. Furthermore, no correlation was observed between MR signal and cell graft survival. All rejected cell grafts retained histologic iron, resulting in a “false positive” indicator of cell survival, which makes drawing conclusions about transplanted cell graft viability from histological iron challenging, as was shown in previous studies 14. However, it is still possible to use the histological iron as an indicator of graft location because it correlated with graft location confirmed with human nuclear staining. More accurate and sensitive quantitative approaches have been successfully used with advanced molecular imaging probes 42 and reporter gene systems 9, but these probes have not yet received FDA approval.

Understanding the long‐term fate of transplanted ferumoxytol nanoparticles (degree of particle coat degradation, exocytosis or retention in labeled cells, uptake by host phagocytes, amount in extracellular space) is important. Previous groups have reported that transplanted cells do not retain the SPION label long term 43, 44. In this present study, iron deposits from SPION were observed outside of transplanted cells, but transplanted cells retained a level of iron oxide, as was observed with human nucleus‐PB costaining and TEM. The percentage of cells retaining SPION significantly declined over time for both hNPC‐F^Low^ and hNPC‐F^High^ grafts, suggesting exocytosis of the SPION, which is in agreement with previous studies 44. Even without the knowledge of the exact fate of the nanoparticles, important conclusions about the location and size of transplanted cell grafts can still be drawn.

As academic and biotechnology teams push to move cell therapeutics through to the clinical scenario, the importance of developing technologies for diagnostic monitoring of transplanted cell therapies becomes imperative. Diagnostic monitoring of transplanted cellular therapeutics will most likely be required by future clinical trials to properly assess accuracy, delivered dose, as well as long‐term effectiveness and safety 45. In addition to the diagnostic capabilities, this approach has the capability to inform surgical implantation procedures through real‐time visualization and guidance 46.

## Conclusion

In this study, we demonstrated the utility of ferumoxytol labeling in diagnosing accuracy and volume of transplanted cell grafts in vivo and confirming graft location postmortem. The method of SPION labeling described here can provide immediate postoperative confirmation of engraftment and allow for quantification of cell delivery and injection accuracy, addressing key limitations in current clinical trials.

## Author Contributions

J.J.L.: conception and design, collection and/or assembly of data, data analysis and interpretation, manuscript writing, final approval of manuscript; J.G. and L.N.U.: collection and/or assembly of data, data analysis and interpretation; C.V.H., E.A., and N.G.: collection and/or assembly of data; C.N.S.: financial support, provision of study material or patients; T.F.: conception and design, financial support, administrative support, collection and/or assembly of data, data analysis and interpretation, final approval of manuscript; J.N.O.: conception and design, administrative support, collection and/or assembly of data, data analysis and interpretation, final approval of manuscript; N.M.B.: conception and design, financial support, administrative support, provision of study material or patients, data analysis and interpretation, final approval of manuscript.

## Disclosure of Potential Conflicts of Interest

J.N.O. is a compensated consultant for Voyager Therapeutics and has compensated research funding from Siemens Medical Solutions. N.M.B. has compensated intellectual property rights with NeuralStem under an exclusive license from Cleveland Clinic; is a compensated consultant for NeuralStem, MRI Interventions, Agilis, and Biomedica; has compensated research funding from ALSA, DOD, NIH, and Tubman Research Institute; and has uncompensated stock options with Boston Scientific, Switch Bio, and Code Runner. The other authors indicated no potential conflicts of interest.

## Supporting information

Supporting Information
