# Supplementary material for: Ferumoxytol Labeling of Human Neural Progenitor Cells for Diagnostic Cellular Tracking in the Porcine Spinal Cord with Magnetic Resonance Imaging
Source: Stem Cells Transl Med. 2016 Aug 29;6(1):139–50. doi: 10.5966/sctm.2015-0422 (PMC5442757; doi:10.5966/sctm.2015-0422)
Supplement: Supplementary file 1 — Supporting Information [file SCT3-6-139-s001.pdf]

Supplemental Figures – Boulis and Lamanna et al.

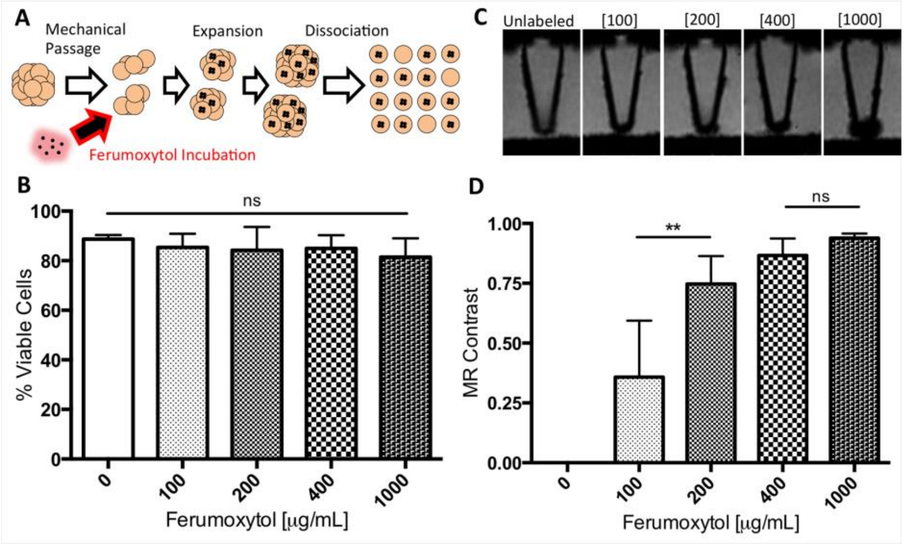

Figure S1. Incubation of human cortical neurospheres with ferumoxytol nanoparticles. Human neural progenitor cells cultured as free floating cortical neurospheres were incubated with increasing concentrations [0 – 1000 µg/mL] of ferumoxytol nanoparticles for 7 days. Following incubation, the neurospheres were washed and chemically dissociated to a single cell suspension (**A**). Results of cell viability assessed by trypan blue exclusion assay with increasing ferumoxytol concentration (**B**). Microcentrifuge tubes with a $2.5x10^6$ cell pellet were immersed in water and imaged with a gradient echo T2*-weighted sequence on a clinical 3T Magnetic Resonance Imaging scanner. Representative images show a signal void in the region of the cell pellet produced by ferumoxytol (**C**). The contrast for the volume of the cell pellet was calculated (**D**). Incubation concentrations of low dose [200] and high dose [400] µg/mL ferumoxytol were chosen for further evaluation and designated hNPC-F^{Low} and hNPC-F^{High}, respectively. These conditions were chosen based on the significant increase in MR contrast between [100] and [200] µg/mL ferumoxytol and the non-significant difference between [400] and [1000]. Graphs displayed as mean ± SD. Abbreviations: MR, Magnetic Resonance; T, Tesla. *Significant, P < 0.05; **Significant, P < 0.005; ***Significant, P < 0.0005.

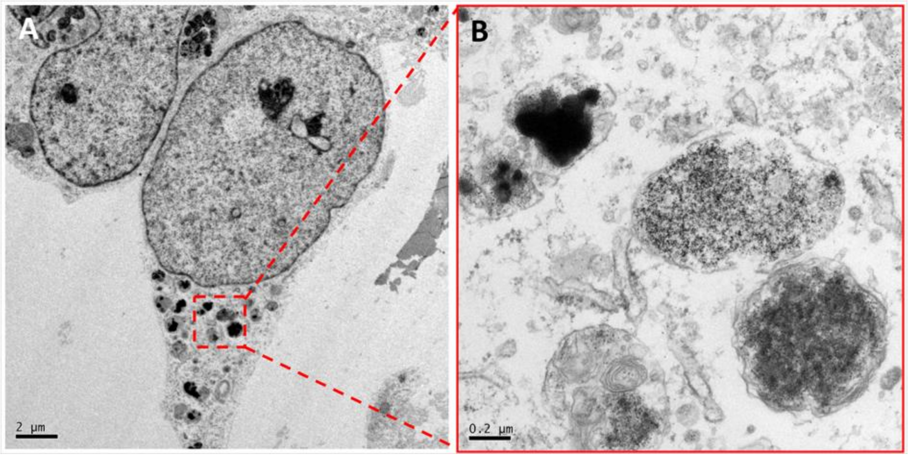

Figure S2. Ferumoxytol nanoparticles observed in labeled human neural progenitor cells with Transmission Electron Microscopy 7 days after labeling. A representative image of transmission electron microscopy of hNPC-F^{High} 7 days after labeling revealed numerous iron-laden, electron-dense endosomes (**A**) containing nanoparticles (**B**). Scale bars: (**a**) 2 µm; and (**b**) 0.2 µm. Abbreviations: hNPC, human neural progenitor cell; F, ferumoxytol.

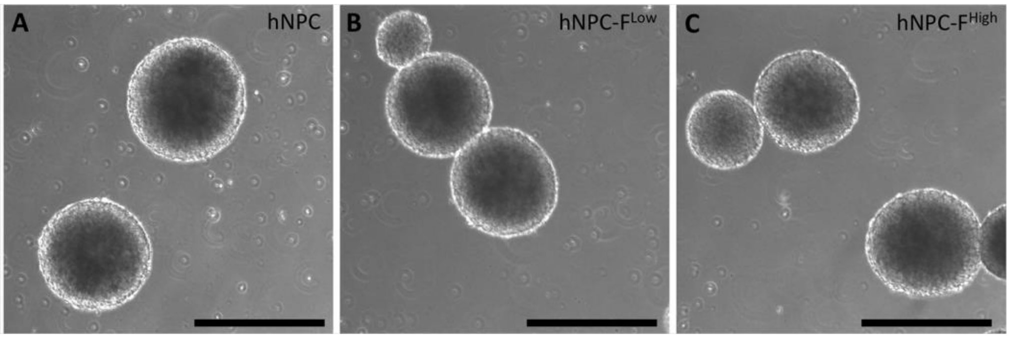

Figure S3. Ferumoxytol-labeled human neural progenitor cells re-form cortical neurospheres. Representative images of hNPC (**a**), hNPC-F^{Low} (**b**), and hNPC-F^{High} (**c**) neurospheres demonstrate healthy morphology following ferumoxytol labeling. The neurospheres were labeled with ferumoxytol using the standard method, but were passaged with the tissue chopper and allowed to re-form neurospheres in cell culture. Scale bar: 4X, 500 µm. Abbreviations: hNPC, human neural progenitor cell; F, ferumoxytol.

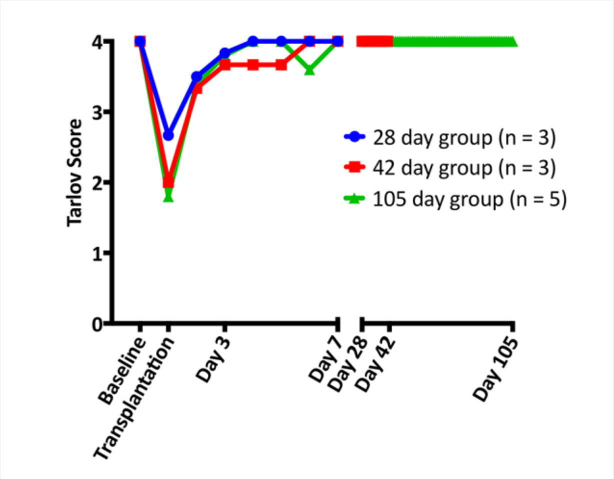

Figure S4. Motor function assessed with the Tarlov Score. The Tarlov score was used to assess gait and motor function in pigs that underwent transplantation. The score is as follows: (0) no voluntary limb function; (1) only perceptible joint movement; (2) active movement but unable to stand; (3) to be able to stand but unable to walk; (4) complete normal hind-limb motor function. Transient morbidity was observed in all groups, but all animals returned to baseline after seven days.

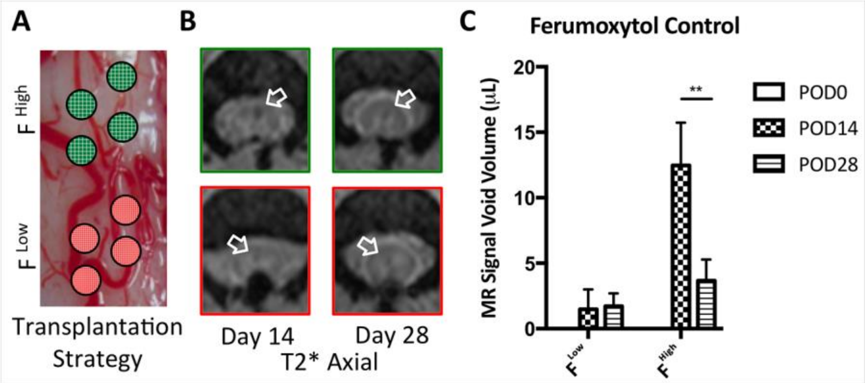

Figure S5. Injection of free ferumoxytol nanoparticles into the porcine spinal cord. Two minipigs received eight 25-µL injections: four high dose ferumoxytol nanoparticle-only injections ($F^{High)}$ into the rostral spinal cord segment and four low dose ferumoxytol nanoparticle-only injections (F^{Low}) into the caudal segment (**A**). Hypointense foci, representative of F^{Low} and F^{High} injections (white arrows) were observed on post-operative day 14 and 28 in representative axial T2*-weighted images (**B**). The injection volumes were calculated for all F^{Low} and F^{High} injections at all time points. Summary data represented as the mean signal intensity at each time point is shown (**C**). Abbreviations: F, ferumoxytol; MR, magnetic resonance; POD, post-operative day; T, tesla. Graphs displayed as mean ± SD. *Significant, $P < 0.05$; **Significant, $P < 0.005$; ***Significant, $P < 0.0005$.

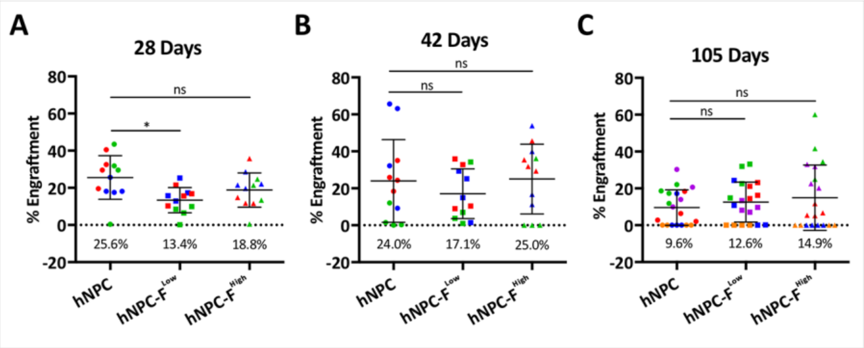

Figure S6. Quantification of transplanted ferumoxytol-labeled human neural progenitor cell graft survival with stereology of human nuclei. Stereological quantification was done for each individual cell graft in each animal for the 28 (**A**), 42 (**B**), and 105 (**C**) day cohorts. Engraftment % is defined as the relative percentage of surviving human cells compared to the amount originally transplanted. Colors of individual data points correspond to individual grafts from individual animals. Abbreviations: hNPC, human neural progenitor cell; F, ferumoxytol. Graphs displayed as mean ± SD. *Significant, P < 0.05; **Significant, P < 0.005; ***Significant, P < 0.0005.

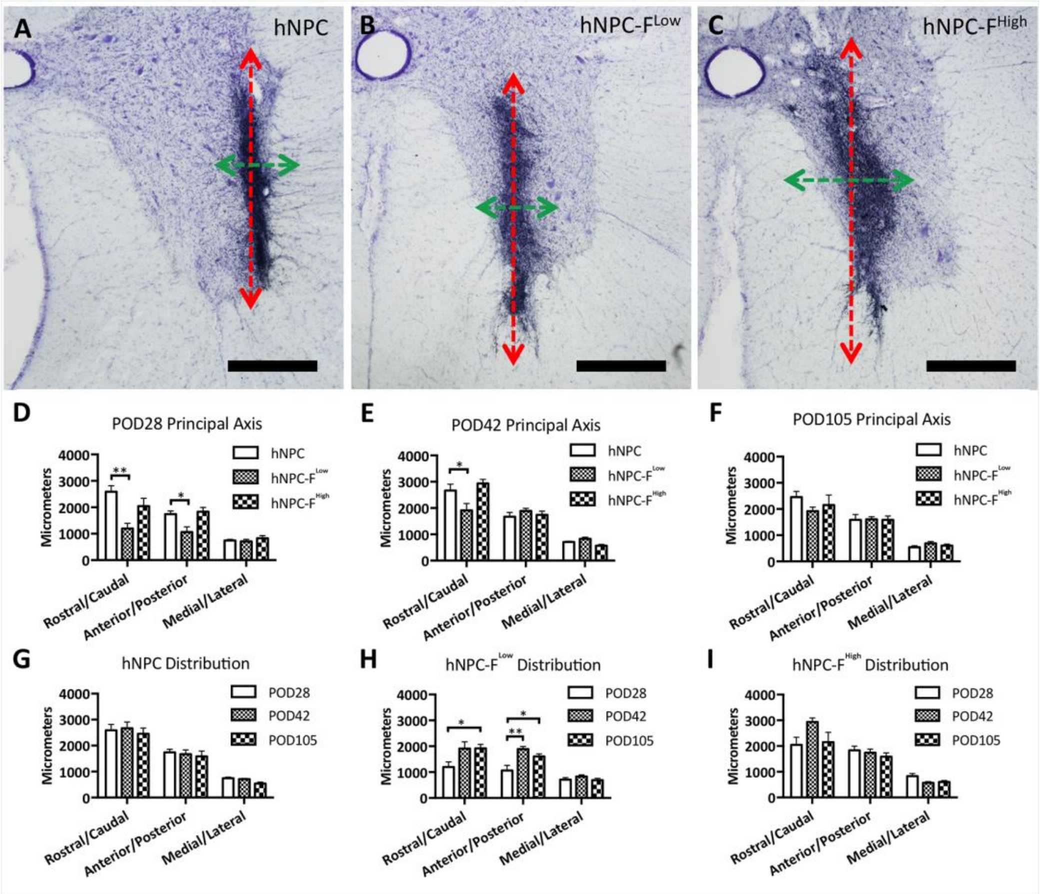

Figure S7. Distribution of ferumoxytol-labeled human neural progenitor cell grafts. Linear measurements were performed along the principle axes (medial/lateral, anterior/posterior, and rostral/caudal) for all non-rejected hNPC, hNPC-F^{Low} and hNPC-F^{High} cell grafts in in the POD28, 42, and 105 day groups (n = 101 grafts total). Representative photomicrographs from hNPC (**A**), hNPC-F^{Low} (**B**) and hNPC-F^{High} (**C**) cell grafts stained for the human nuclear antigen (black nuclei) in the 105 day group show measurements in the anterior/posterior (red) and medial/lateral (green) axes. Quantitative data is shown comparing hNPC, hNPC-F^{Low} and hNPC-F^{High} cell graft distribution for 28 (**D**), 42 (**E**), and 105 (**F**) day cohorts. Data is shown comparing distribution over time for hNPC (**G**), hNPC-F^{Low} (**H**), and hNPC-F^{High} (**I**) cell grafts. Measurements were done using ImageJ. Abbreviations: F, ferumoxytol; hNPC, human neural progenitor cell; POD, post-operative day. Graphs displayed as mean ± SD. Scale bars: 4X, 500 µm. *Significant, $P < 0.05$; **Significant, $P < 0.005$; ***Significant, $P < 0.0005$.

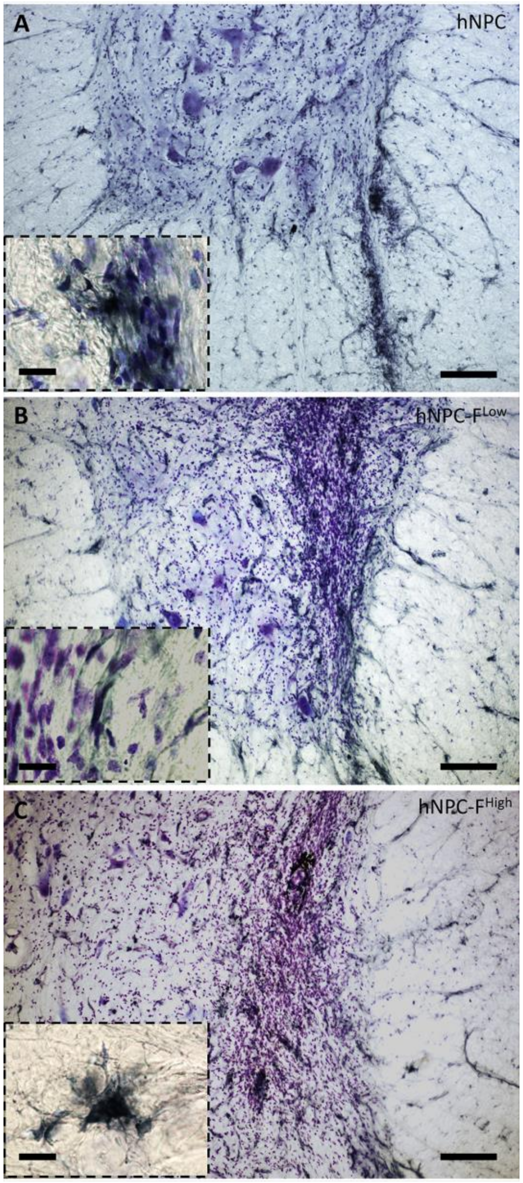

Figure S8. Microglial activation following transplantation of human neural progenitor cells. Representative micrographs of immunohistochemical staining with a rabbit polyclonal anti-Iba1 antibody to assess microglial activation are shown for unlabeled hNPC (**A**), hNPC-F^{Low} (**B**), and hNPC-F^{High} (**C**) cell grafts from post-operative day 105 with cresyl violet background stain. Five grafts from each condition with >10% engraftment were chosen for assessment. Scale bars: 10X, 200 µm; inserts 100X, 25 µm. Abbreviations: F, ferumoxytol; hNPC, human neural progenitor cell.
